# Enhanced Bone Regeneration in Variable-Type Biphasic Ceramic Phosphate Scaffolds Using rhBMP-2

**DOI:** 10.3390/ijms222111485

**Published:** 2021-10-25

**Authors:** Ho-Kyung Lim, Ik-Jae Kwon, Sung-Woon On, Seok-Jin Hong, Byoung-Eun Yang, Soung-Min Kim, Jong-Ho Lee, Soo-Hwan Byun

**Affiliations:** 1 Department of Oral and Maxillofacial Surgery, Korea University Guro Hospital, Seoul 08308, Korea; ungassi@naver.com; 2 Department of Oral & Maxillofacial Surgery, School of Dentistry, Seoul National University, Seoul 03080, Korea; ijkwon@snu.ac.kr (I.-J.K.); smin5@snu.ac.kr (S.-M.K.); leejongh@snu.ac.kr (J.-H.L.); 3 Department of Oral and Maxillofacial Surgery, Dentistry, Dongtan Sacred Heart Hospital, Hallym University College of Medicine, Hwaseong 18450, Korea; drummer0908@hanmail.net; 4 Department of Otolaryngology-Head & Neck Surgery, Dongtan Sacred Heart Hospital, Hallym University College of Medicine, Hwaseong 18450, Korea; enthsj@hanmail.net; 5 Department of Oral and Maxillofacial Surgery, Dentistry, Sacred Heart Hospital, Hallym University College of Medicine, Anyang 14068, Korea; face@hallym.or.kr; 6 Graduate School of Clinical Dentistry, Hallym University, Chuncheon 24252, Korea; 7 Research Center of Clinical Dentistry, Clinical Dentistry Graduate School, Hallym University, Chuncheon 24252, Korea

**Keywords:** biphasic calcium phosphate, block-type biphasic calcium phosphate scaffold, collagen, recombinant human bone morphogenic protein 2, bone regeneration

## Abstract

Our aim was to investigate the bone regeneration capacity of powder-type biphasic ceramic scaffold (BCP powder), block-type BCP (BCP block), and collagen-added block-type BCP (BCP collagen) with different concentrations of recombinant human bone morphogenetic protein 2 (rhBMP-2) in an animal model. Four rabbits were assigned to each of the following groups: no graft + rhBMP-2 (0.1/0.2 mg/mL), BCP powder + rhBMP-2 (0.1/0.2 mg/mL), BCP block + rhBMP-2 (0.1/0.2 mg/mL), and BCP collagen + rhBMP-2 (0.1/0.2 mg/mL), i.e., a total of 32 rabbits. Polycarbonate tubes (Φ 7 mm × 5 mm) for supporting scaffolds were fixed into a 7 mm round border. Subsequently, 0.1 mL of rhBMP-2 solutions with different concentrations was injected into the tubes. Both radiological and histomorphometric analyses showed that osteogenesis was not enhanced by increasing the concentration of rhBMP-2 in all groups at both 3 and 6 weeks. Radiological analysis showed that bone formation was higher in the BCP collagen group than in the BCP powder and BCP block groups at both rhBMP-2 concentrations at 3 weeks. rhBMP-2 enhanced bone formation; however, as the concentration increased, bone formation could not be enhanced infinitely. Collagen-added alloplastic graft material may be useful for mediating rapid bone formation in initial stages.

## 1. Introduction

Selection of an appropriate bone graft material is necessary for ensuring successful alveolar bone augmentation. Autogenous bone grafts are considered the gold standard treatment; however, donor site morbidity limits their application, and it is difficult to provide a sufficient amount [1]. Therefore, tissue engineering technologies have been developed for bone augmentation, and many studies have focused on the development and application of growth factors and bone substitute materials [2,3]. Tissue engineering involves the new generation of tissues from cells with certain scaffolds and signaling molecules [4]. Bone marrow-derived mesenchymal stem cells have been studied for various clinical applications, especially for bone regeneration [5,6]. Mesenchymal stem cells can be obtained using a minimally invasive technique, and their use can obviate the need for donor site surgery in autogenous bone graft procedures [6].

Recombinant human bone morphogenetic protein (rhBMP) is the most studied bone growth factor [7]. In particular, rhBMP-2 has received considerable attention for enhancing bone augmentation [8,9]; the capacity for osteoinduction allows its use as an alternative approach to autogenous bone grafts in bone defect sites [8,10]. However, since rhBMP-2 shows high solubility, it is rapidly dissolved in the absence of an appropriate delivery system [11]. Therefore, in most cases, rhBMP-2 is deposited on an effective scaffold [12]. In addition, studies on the optimal concentration of rhBMP-2 for inducing bone formation without side effects are still in progress [13].

Currently, biphasic calcium phosphate (BCP), a synthetic bone substitute material, is widely used as a carrier for rhBMP-2; it comprises a mixture of hydroxyapatite (HA) and tricalcium phosphate (TCP) [14,15]. BCP resembles the inorganic phase of the human bone tissue [14]. Beta-TCP is an absorbable ceramic that directly forms bonds with the bone [16]. β-TCP granules are rapidly dissolved and filled with newly formed bone tissue [16]. HA is combined with β-TCP to prevent resorption of the structure before the new bone is filled [17,18]. This improves the formation and remodeling of the new bone by providing an appropriate resistance [17]. The use of BCP as a carrier of rhBMP-2 produces good results, and the use of BCP grafts combined with a growth factor may be biocompatible and histologically successful in forming new bones [19,20].

Since powder-type BCPs have high versatility, they are widely used in actual clinical practice [21]. However, it is difficult to apply BCP to the site-loaded external compressive force since its shape may collapse [21]. In addition, since the potency of growth factors depends on many factors, such as the composition of the scaffold, pore size, geometrical form, and porosity, the need to increase the preservation capacity of growth factors and expanding clinical indications have emerged via improvement of the physical form of BCP [22].

The use of block-type BCP for improving the versatility of powder-type BCPs is advantageous in terms of volume maintenance and stability. Block-type BCP possesses desirable mechanical properties to be grafted at a large defect site [23]. However, block-type BCPs have the disadvantages of being difficult to manipulate and are brittle, and there may be restrictions in supporting growth factors depending on pore size or porosity [23]. Therefore, to increase the preservation of growth factors, a recent study has attempted to add collagen to BCP [24]. When collagen is added to BCP, although it can disassemble when hydrated, it has a stronger physical cohesion than that of the powder type; therefore, application to the surgical site is relatively easier, and this application increases the potency of growth factors [25]. However, certain clinicians show concern regarding the early resorption of collagen-added BCP due to the properties of collagen. For these reasons, various studies have been performed to determine the ideal grafting material and method.

In this study, the mechanical strength of block-type and collagen-added BCPs was investigated, and the bone formation ability of powder-type (BCP powder), block-type (BCP block), and collagen-added (BCP collagen) BCPs was compared in a rabbit model. Furthermore, the differences in bone formation ability according to rhBMP-2 concentration were evaluated. The purpose of this study was to determine the optimal protocol for enhancing bone regeneration at the early or late stages after grafting alloplastic bone with rhBMP-2.

## 2. Results

### 2.1. Compression Test of Block Scaffold

The compressive strength of the BCP block was 1.35 ± 0.04 MPa. The compressive strength of BCP collagen was 1.11 ± 0.11 MPa.

### 2.2. Clinical Findings in Animal Experiments

No specific clinical features were found in the experimental specimens during the study period. No specific findings such as abnormal behavior, infection, wound dehiscence, or edema were observed. No morphological abnormalities or pathological findings were observed at the surgical site, even at three and six weeks from euthanasia.

### 2.3. Radiological Analysis

**Figure 1:**
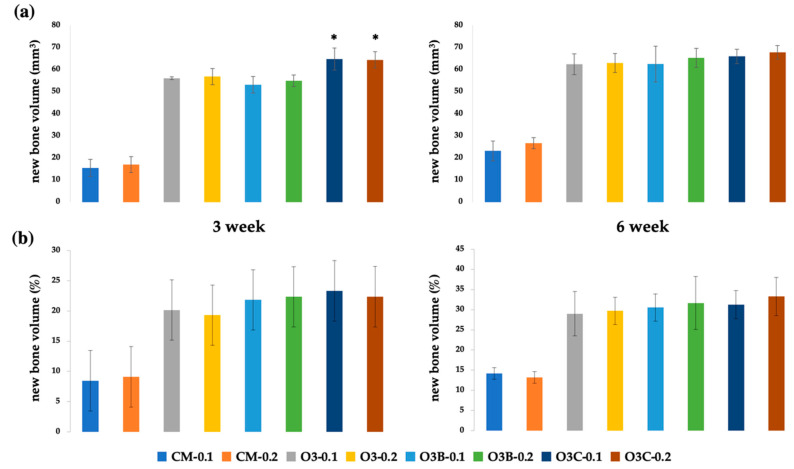
Evaluation of bone formation via radiological analysis and histomorphometric analysis. (**a**) radiological analysis; (**b**) histomorphometric analysis. rhBMP-2, recombinant human bone morphogenetic protein 2; BCP, biphasic ceramic phosphate scaffold; *, At the same concentration of rhBMP-2, bone formation occurred to a greater extent in BCP collagen than in BCP block and BCP powder at 3 weeks POD (*p* < 0.05); CM-0.1, no graft containing 0.1 mg/mL rhBMP-2; CM-0.2, no graft containing 0.2 mg/mL rhBMP-2; O3-0.1, BCP powder containing 0.1 mg/mL rhBMP-2; O3-0.2, BCP powder containing 0.2 mg/mL rhBMP-2; O3B-0.1, BCP block containing 0.1 mg/mL rhBMP-2; O3B-0.2, BCP block containing 0.2 mg/mL rhBMP-2; O3C-0.1, collagen-added BCP containing 0.1 mg/mL rhBMP-2; O3C-0.2, collagen-added BCP containing 0.2 mg/mL rhBMP-2.

**Figure 2:**
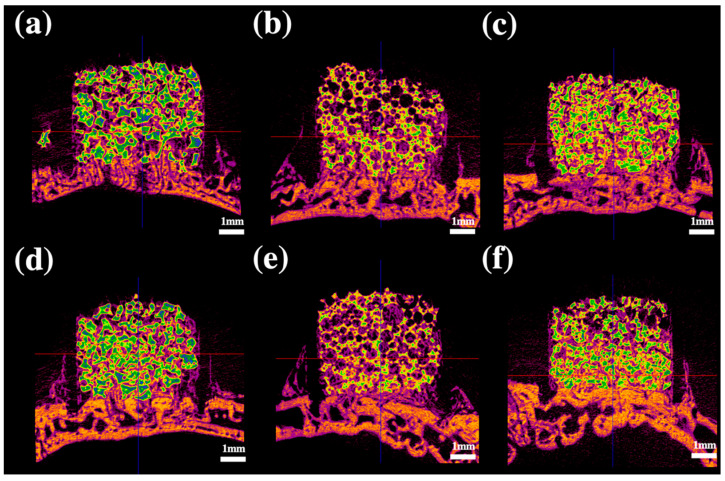
Radiological analysis after 3 weeks. (**a**) BCP powder containing 0.1 mg/mL rhBMP-2; (**b**) BCP block containing 0.1 mg/mL rhBMP-2; (**c**) collagen-added BCP containing 0.1 mg/mL rhBMP-2; (**d**), BCP powder containing 0.2 mg/mL rhBMP-2; (**e**), BCP block containing 0.2 mg/mL rhBMP-2; (**f**), collagen-added BCP containing 0.2 mg/mL rhBMP-2; BCP, biphasic ceramic phosphate scaffold; rhBMP-2, recombinant human bone morphogenetic protein 2.

Radiographic analysis at 3 and 6 weeks POD (post operative day) showed that bone formation induced by BCP powder, BCP block, and BCP collagen was higher than that observed in the absence of grafts at the same concentration of rhBMP-2 (*p* < 0.05). Bone formation increased in each group over time (*p* < 0.05) (Figure 1). At the same concentration of rhBMP-2, bone formation occurred to a greater extent in BCP collagen than in BCP block and BCP powder at 3 weeks POD (*p* < 0.05) (Figure 2). However, there was no significant difference in bone formation between BCP powder, BCP block, and BCP collagen at the same concentration of rhBMP-2 at 6 weeks POD (*p* > 0.05). Bone formation was not altered in BCP collagen compared to that in BCP block and BCP powder at 6 weeks POD (*p* > 0.05). There was no difference in bone formation based on the concentration of rhBMP-2 at 3 and 6 weeks POD (*p* > 0.05).

### 2.4. Histomorphometric Analysis

**Figure 3:**
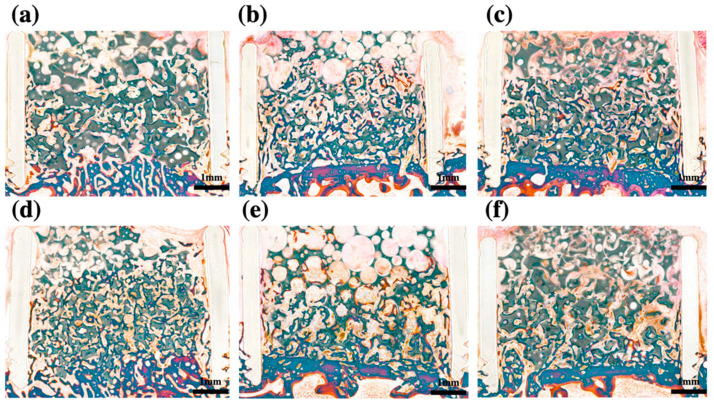
Histomorphometric analysis after 3 weeks. (**a**) BCP powder containing 0.1 mg/mL rhBMP-2; (**b**) BCP block containing 0.1 mg/mL rhBMP-2; (**c**) collagen-added BCP containing 0.1 mg/mL rhBMP-2; (**d**), BCP powder containing 0.2 mg/mL rhBMP-2; (**e**), BCP block containing 0.2 mg/mL rhBMP-2; (**f**), collagen-added BCP containing 0.2 mg/mL rhBMP-2; BCP, biphasic ceramic phosphate scaffold; rhBMP-2, recombinant human bone morphogenetic protein 2.

Histomorphometric analysis at 3 and 6 weeks POD showed that bone formation induced by BCP powder, BCP block, and BCP collagen was higher than that observed in the absence of grafting (*p* < 0.05) at the same concentration of rhBMP-2. Bone formation increased in each group over time (*p* < 0.05). There was no significant difference in bone formation between BCP powder, BCP block, and BCP collagen at the same concentration of rhBMP-2 at both 3 and 6 weeks POD (*p* > 0.05). Bone formation was not altered in BCP collagen compared to that in BCP block and BCP powder at both 3 and 6 weeks POD (*p* > 0.05) (Figure 1). There was no difference in bone formation based on the concentration of rhBMP-2 (*p* > 0.05) (Figure 3).

## 3. Discussion

In this study, the compressive strengths of BCP block and BCP powder were 1.35 ± 0.04 and 1.11 ± 0.11 MPa, respectively. Previous studies report that the great compressive strength of mandibular cancellous bone in normal humans is approximately 3.9 MPa, and that of mandibular cortical bone is 23.7, 21.6, and 19.6 MPa in the anterior, premolar, and molar regions, respectively [26,27]. The iliac bone, which is frequently used as a harvesting site for autologous block bone grafts, has a compressive strength of approximately 13.58 MPa [28]. Both BCP block and BCP powder showed lower compressive strengths than those of the cancellous bone, cortical bone, and iliac bone. An improvement in strength of BCP block is considered necessary for increasing clinical application.

BCP comprises HA and β-TCP at various ratios. BCP powder, block, and collagen used in this study consisted of 60% HA and 40% β-TCP, and contained particles of 0.5–1.0 mm in diameter. β-TCP is an absorbent ceramic and has a faster absorption rate than that of HA in the body and provides a space to be replaced by the new bone [29]. HA has a slower absorption rate than that of β-TCP and helps maintain the structure of the scaffold until the new bone is formed [17]. In previous research, it was reported that new bone formation is increased when the β-TCP ratio is 70% compared to when it is 30% [30]. However, since dimensional shrinkage increases as the proportion of β-TCP increases in BCP, a high β-TCP/HA ratio is detrimental to the mechanical stability of the scaffold [31]. In addition, BCP with a higher proportion of HA is considered more appropriate for use as rhBMP carriers compared to BCP containing a lower HA proportion [32]. Therefore, a scaffold with an HA/TCP ratio of 6:4 is considered optimal and was used in this study.

The extent of new bone formation may differ depending on the pore size or porosity of the scaffold. An optimal pore size or porosity has not been established previously. It has been reported that a small pore size in the scaffold may be detrimental to initial bone formation [33]; a study reported enhanced bone regeneration when the pore size is >300 μm [34]. Similar to the pore size, it has been reported that the higher the porosity value, the better the bone formation [35]. However, an increase in pore size may be disadvantageous to vascularization and the ability to store growth factors is reduced. In addition, since the mechanical stability of the scaffold decreases as the porosity increases, the appropriate pore size and porosity of the scaffold represent important factors. Although pore size and porosity were not compared in this study, normal bone formation was observed using BCP scaffolds.

Several studies have confirmed that the block-type BCP is efficient in maintaining space [36]. Although alloplastic block bone shows lower bone formation than that obtained using autologous block bone [37], the former shows superior bone formation compared to that observed in the absence of grafts [36]; additionally, it has been reported that greater bone formation is observed when rhBMP-2 is added [38]. In addition, the block-type BCP exhibits superior bone regeneration ability compared to that of the particle type [39], and the use of block-type BCP is reported to be advantageous for barrier membrane support [40]. The block-type scaffold has greater mechanical strength to resist the outer force than that of the powder-type scaffold. This property can enhance bone formation under force-bearing conditions. However, in this study, the BCP block did not show higher bone regeneration than that observed using BCP powder. This result may be attributed to polycarbonate tubes, which can support the outer force. The tube might have diminished the difference in the mechanical properties between the BCP block and BCP powder. Due to the surrounding polycarbonate tubes used in this study, the bone formation or resorption of the grafted scaffold may be altered in clinical applications.

Radiological analysis showed that a relatively higher bone regeneration ability was observed when collagen was added at an early stage (3 weeks POD). Viscosity and wettability that are increased by the addition of collagen enabled easier manipulation and enhanced the storage of the rhBMP-2 solution during actual clinical application [41,42]. Due to these characteristics of collagen, BCP collagen composites show a superior bone forming capacity in the early stage [43]. Collagen presentation of BCP can affect the mineralization of bone grafts when compared with the pure BCP graft [44]. The change in graft composition according to the addition of collagen affects its solubility and differentiation of osteoclast precursor cells into mature osteoclasts [45]. A previous study showed that cell affinity and alkaline phosphatase activity increase when collagen is coated on the surface of BCP [46]. In addition, collagen affects the carrying capacity of the growth factors. In fact, other studies have shown that various types of collagen, such as sponges and membranes, play an excellent role as rhBMP-2 carriers [47]. This may affect the rapid bone formation of BCP collagen at an early stage compared to that in the non-collagen BCP scaffold.

No disagreement has been reported regarding the positive effect of rhBMP-2 on bone formation; however, the difference in bone formation performance according to the dose of rhBMP-2 is controversial. In several studies, the dose-dependent efficiency of rhBMP-2 carriers has been shown to differ [20,48,49]. However, it was also reported that it is impossible to observe the difference in bone formation according to the concentration of rhBMP-2 [50]. Our study showed that there was no significant difference in bone formation between the rhBMP-2 concentrations of 0.1 mg/mL and the 0.2 mg/mL. Considering the pharmacokinetic differences between animals and humans, it is considered that it will be very difficult to identify the appropriate concentration of rhBMP-2; however, it is expected that consensus on the appropriate concentration will be reached someday.

The limitations of this study include the lack of sample size, short observation period, and absence of cellular tests. However, it was demonstrated that the shape of the scaffold and the addition of collagen can affect bone formation in the early stage of graft. The findings of this study will assist clinicians choosing the effective scaffold.

## 4. Materials and Methods

### 4.1. Preparation of Ceramic Scaffolds

**Figure 4:**
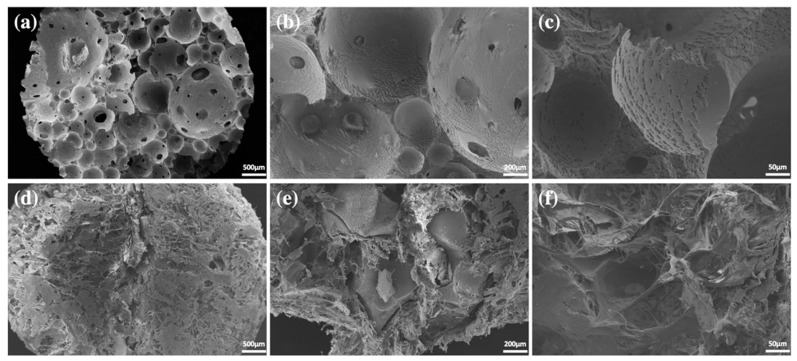
Microscopic structure of block-type and collagen-added BCPs determined via scanning electron microscopy. (**a**) Block-type BCP image (×100). (**b**) Block-type BCP image (×300). (**c**) Block-type BCP image (×1000). (**d**) Collagen-added BCP image. BCP, biphasic ceramic phosphate scaffold (×100). (**e**) Collagen-added BCP image. BCP, biphasic ceramic phosphate scaffold (×300). (**f**) Collagen-added BCP image. BCP, biphasic ceramic phosphate scaffold (×1000).

Three types of BCP scaffolds were prepared. The first was BCP powder without collagen (OSTEON 3, Genoss^®^, Suwon, Korea). The HA/β-TCP ratio in this scaffold was 60:40, and the calcium/phosphorus ratio was in the range of 1.5–2.0. Particle size ranged from 0.5 to 1.0 mm, and the porosity of the particles was less than 80%. The macropore size was 200–400 µm, and the micropore size was <10 µm. The second BCP was BCP block without collagen (OSTEON 3 block, Genoss^®^). BCP block was manufactured using the direct foaming method, and the HA/TCP and Ca/P ratios were the same as those of BCP powder. BCP block was manufactured in a cylindrical shape with a diameter of 7 mm and a height of 5 mm to fit a critical-sized defect (CSD) that was created during animal experiments. The third BCP comprised BCP collagen that contained type 1 collagen (OSTEON 3 collagen, Genoss^®^). The HA/TCP ratio, Ca/P ratio, and powder size were the same as those of the collagen-free formulation, and the collagen content was 8% (Figure 4).

### 4.2. Compression Test of Block-Type Scaffold

The compression strengths of the BCP block and BCP collagen specimens were measured. The cylindrical specimens of 7 mm diameter and 5 mm height, manufactured in the same manner as for animal experiments, were placed in a universal testing apparatus (H50K-T UTM^®^, Tinius Olsen Corp., Surrey, UK), and the compressive load forced on the specimen was evaluated by lowering the rod at a rate of 0.5 ± 0.1 mm/min. The distance (μm) was measured against the stress (N/cm^2^, log scale). The compressive load values were determined by identifying the intersection area where the load increased rapidly, and the stress increased gradually in the graph. A total of 30 tests were repeated to obtain descriptive statistics.

### 4.3. Animal Experiments

**Figure 5:**
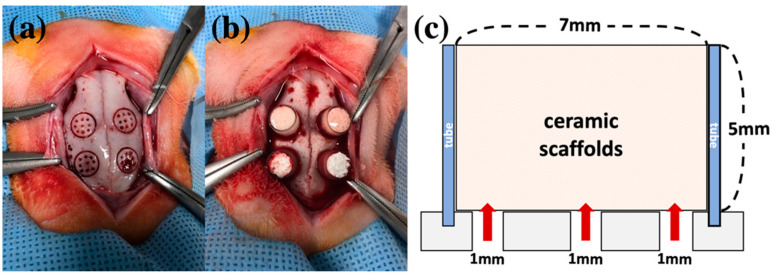
Protocol of animal experiments. (**a**) Four defects in calvarial area. A round border of 7 mm in diameter with nine holes that were 1 mm in diameter were created. (**b**) Application of scaffold into polycarbonate tubes (Φ 7 × 5 mm). (**c**) Schematic of the animal experiment.

Animal experiments were performed with the approval of the Animal Ethics Committee of Genoss Laboratory (Animal Laboratory Approval No: GEN-IACUC-1803-02; Date: 19 March 2018) and according to the ARRIVE and PREPARE guidelines [51,52]. To observe statistical differences in bone formation between scaffolds, as well as in the stability and blood flow of scaffolds, rabbit calvaria defects were selected as an animal model for our experiments. The experiments were performed using a total of 32 male New Zealand white rabbits (age, 7 weeks; weight, 2.5 ± 0.5 kg). Based on the scaffold type, the concentration of rhBMP-2, and sacrifice timing, the rabbits were assigned to the following 16 groups (two individuals with eight CSDs per group): no graft + rhBMP-2 (0.1/0.2 mg/mL), BCP powder + rhBMP-2 (0.1/0.2 mg/mL), BCP block + rhBMP-2 (0.1/0.2 mg/mL), and BCP collagen + rhBMP-2 (0.1/0.2 mg/mL). Surgery was performed under intravenous injection of general anesthesia using xylazine HCl (10 mg/kg; Rompun^®^, Bayer, Korea, Seoul, Korea) and a mixture of tiletamine and zolazepam (50 mg/kg; Zoletil^®^, Virbac Korea, Seoul, Korea). After shaving the forehead area, the area was disinfected using betadine, and lidocaine with 1:100,000 epinephrine was injected into the incision area to reduce pain and bleeding. After exposing the forehead skull via incision and periosteal dissection, a total of 4 CSDs (7 mm diameter) were formed in the calvarial area. Multiple holes with a diameter of 1 mm were formed in the CSD for ensuring blood supply to the scaffold [53]. Polycarbonate tubes (Φ 7 × 5 mm) for supporting scaffolds were fixed into a 7 mm round border, and the scaffold was inserted into the tube according to the study design [54]. Subsequently, 0.1 mL of rhBMP-2 solution (BMP, Genoss^®^, Suwon, Korea) was injected into the tube according to the study design (Figure 5). To prevent the scaffold from falling off, the tube was covered using a cap, and a layer-to-layer interrupted suture was performed using 4–0 Vicryl^®^ (Johnson & Johnson, Brunswick, GA, USA). Gentamicin sulfate (5 mg/kg; intramuscular, Sinil, Seoul, Korea) and diclofenac (5 mg/kg, IM, Sinil, Seoul, Korea) were administered for 3 d after surgery to prevent infection and as an analgesic. Inflammation, wound dehiscence, general health, and infection were monitored daily. The occurrence of inflammation, wound dehiscence, infection, abnormal behavior of individuals, and uncontrolled bleeding were exclusion criteria. Thereafter, 16 animals were euthanized at the 3rd and 6th weeks of the postoperative period. After general anesthesia was induced using xylazine HCl, tiletamine, and zolazepam, euthanasia was performed by injecting potassium chloride into the ear vein. The calvarium area where the scaffold was implanted was excised after euthanasia. The harvested specimens were fixed in 10% formalin solution.

### 4.4. Radiological Analysis

The skull was loaded on a micro-computed tomography system (SkyScan1173^®^, Ver. 1.6; Bruker-CT, Kontich, Belgium), and sliced images were obtained. The shooting was performed using a tube current of 60 μA, tube voltage of 130 kVp, aluminum filter of 1 mm, rotation angle of 0.3°, and exposure time of 500 ms. High-resolution images were acquired containing 2240 × 2240 pixels and the pixel size was 13.85 μm. The captured images were recombined using NRecon (Ver. 1.7; Bruker-CT, Kontich, Belgium), and three-dimensional reconstruction of the recombined images was performed using Ct-VOX (Ver. 1.14.4.1; Bruker-CT, Kontich, Belgium). In the program, the new bone volume was calculated according to the following Equation (1):

Percent bone volume (%) = new bone volume/total volume × 100(1)

### 4.5. Histological Analysis

The excised tissue was prepared as a non-decalcified specimen. For a period of approximately 1 week, the excised tissue was immersed in formalin solution and fixed. Subsequently, the specimen was washed in running water for 12 h, and dehydration was performed using ethanol, which was sequentially increased in concentration from 70% to 100%. Technovit 7200 resin (Heraeus KULZER, Hanau, Germany) was infiltrated into the tissue, and the process was performed for 2 d to enable complete penetration of the resin. The resin-embedded tissue was cured using a UV Embedding system (KULZER EXAKT 520^®^, Heraeus KULZER, Hanau, Germany), and a cross-section of the material and bone tissue was obtained by cutting the fabricated resin block. The prepared slides were stained with Goldner’s trichrome stain. Images of the tissue slides were acquired using an optical microscope (Olympus BX50^®^; Olympus Optical Co., Tokyo, Japan) equipped with a digital image sensor. Subsequently, the AOI (Area of Interest) tool of the Image-Pro Plus^®^ analysis software (Media Cybernetics, Rockville, MD, USA) was used to calculate the area of new bone observed on the tissue slide as following Equation (2):

New bone (%) = area of new bone/total area of defect × 100 (2)

### 4.6. Statistical Analysis

Three statistical analyses were performed. Mann–Whitney U tests were performed to compare differences in rhBMP-2 concentrations within the same scaffold. The Kruskal–Wallis test was performed to compare differences between scaffolds within the same rhBMP-2 concentration. Finally, the Wilcoxon signed-rank test was performed to compare differences during the observation period within the same group. Values of *p* < 0.05 were considered significant; SPSS 23 for Windows (SPSS Inc., Chicago, IL, USA) was used for statistical analysis.

## 5. Conclusions

Administration of rhBMP-2 promoted bone formation; however, a difference in bone formation based on concentration could not be identified. BCP in block form or with collagen supplementation may be more advantageous for bone regeneration in the early stage of bone grafting compared to using BCP powder. Collagen-mixed alloplastic graft material may be useful for inducing rapid bone formation at the initial stage.

## Data Availability

No new data were created or analyzed in this study. Data sharing is not applicable to this article.
